# Does Timepoint of Surgical Procedure Affect the Outcome in Simultaneous Pancreas–Kidney Transplantation? A Retrospective Single-Center Analysis over 20 Years

**DOI:** 10.3390/jcm13133688

**Published:** 2024-06-25

**Authors:** Hans Michael Hau, Nora Jahn, Christos Vlachos, Tim Eichler, Andri Lederer, Antonia Geisler, Uwe Scheuermann, Daniel Seehofer, Sylvia Köppen, Sven Laudi, Robert Sucher, Sebastian Rademacher

**Affiliations:** 1 Department of General-, Visceral- and Transplant Surgery, Medical University of Graz, 8010 Graz, Austria; hans.hau@medunigraz.at (H.M.H.); tim.eichler@stud.medunigraz.at (T.E.); andri.lederer@uniklinikum.kages.at (A.L.); antonia.geisler@medunigraz.at (A.G.); robert.sucher@medunigraz.at (R.S.); 2 Department of Visceral, Transplantation, Vascular and Thoracic Surgery, University Hospital of Leipzig, Liebigstrasse 20, 04103 Leipzig, Germany; christos.vlachos83@outlook.com (C.V.); uwe.scheuermann@medizin.uni-leipzig.de (U.S.);; 3 Department for Anesthesiology and Intensive Care Medicine, Medical University of Graz, 8010 Graz, Austria; nora.jahn@medunigraz.at; 4 Department of Anesthesiology and Intensive Care Medicine, University Hospital of Leipzig, 04103 Leipzig, Germany; sylvia.koeppen@medizin.uni-leipzig.de (S.K.);

**Keywords:** simultaneous pancreas–kidney transplantation, risk factors, surgical complications, sleep deprivation, nighttime procedure

## Abstract

**Background**: Sleep deprivation and disturbances in circadian rhythms may hinder surgical performance and decision-making capabilities. Solid organ transplantations, which are technically demanding and often begin at uncertain times, frequently during nighttime hours, are particularly susceptible to these effects. This study aimed to assess how transplant operations conducted during daytime versus nighttime influence both patient and graft outcomes and function. **Methods**: simultaneous pancreas–kidney transplants (SPKTs) conducted at the University Hospital of Leipzig from 1998 to 2018 were reviewed retrospectively. The transplants were categorized based on whether they began during daytime hours (8 a.m. to 6 p.m.) or nighttime hours (6 p.m. to 8 a.m.). We analyzed the demographics of both donors and recipients, as well as primary outcomes, which included surgical complications, patient survival, and graft longevity. **Results**: In this research involving 105 patients, 43 SPKTs, accounting for 41%, took place in the daytime, while 62 transplants (59%) occurred at night. The characteristics of both donors and recipients were similar across the two groups. Further, the rate of (surgical) pancreas graft-related complications and reoperations (daytime 39.5% versus nighttime 33.9%; *p* = 0.552) were also not statistically significant between both groups. In this study, the five-year survival rate for patients was comparable for both daytime and nighttime surgeries, with 85.2% for daytime and 86% for nighttime procedures (*p* = 0.816). Similarly, the survival rates for pancreas grafts were 75% for daytime and 77% for nighttime operations (*p* = 0.912), and for kidney grafts, 76% during the day compared to 80% at night (*p* = 0.740), indicating no significant statistical difference between the two time periods. In a multivariable model, recipient BMI > 30 kg/m^2^, donor age, donor BMI, and cold ischemia time > 15 h were independent predictors for increased risk of (surgical) pancreas graft-related complications, whereas the timepoint of SPKT (daytime versus nighttime) did not have an impact. **Conclusions**: The findings from our retrospective analysis at a big single German transplant center indicate that SPKT is a reliable procedure, regardless of the start time. Additionally, our data revealed that patients undergoing nighttime transplants have no greater risk of surgical complications or inferior results concerning long-term survival of the patient and graft. However, due to the small number of cases evaluated, further studies are required to confirm these results.

## 1. Introduction

Insufficient sleep is recognized as a risk factor affecting the abilities and efficacy of healthcare practitioners [1]. Controlled studies have confirmed that lack of sleep adversely affects neurocognitive and psychomotor abilities (employing tasks relevant to clinical scenarios, albeit artificially created) and in actual clinical practice and settings [2,3]. As a result, longer working hours, disturbances in natural sleep cycles, and doctor exhaustion are associated with an increased rate of avoidable medical errors [4]. This is particularly significant in the field of surgery, given that existing literature indicates a correlation between sleep deprivation and elevated rates of surgical complications. Many research findings have indicated that surgical complications rank as the second leading cause of preventable illness and death [5,6]. To reduce medical mistakes in surgery due to fatigue, improve patient safety, and raise the standard of healthcare, restrictions on the working hours of physicians have been established in both the European Union and the United States (US) [7,8]. Furthermore, heightened apprehension regarding potential risks associated with overnight procedures has prompted a growing trend toward meticulous examination to determine which surgeries can be safely postponed, altering the transition from nighttime to daytime schedules [9]. Consequently, it would be reasonable to postpone any non-emergency surgical procedures planned for overnight to the next day. 

Nevertheless, solid organ transplants seem particularly susceptible due to the convergence of intricate surgical techniques with an uncertain initiation period, often occurring at night. 

In most cases, this depends on the timing of the time of donor death.

Delaying the retrieval of organs from a brain-dead donor is constrained by both medical and logistical considerations. Postponing the donor procedure increases the likelihood of cardiocirculatory instability, potentially compromising the viability of the organs. This approach appears paradoxical in a time when a growing number of expanded criteria are being embraced due to organ scarcity. Given these considerations and the associated risks of nighttime surgeries in solid organ transplantation, where cold ischemia time (CIT) is a key determinant of both outcome and graft functionality, it is not currently practical to merely postpone the start time of the operation. In this particular domain, numerous studies have indicated that in pancreas transplantation, both prolonged and elevated CIT are linked with higher occurrences of delayed graft function (DGF), episodes of acute rejection, and an increase in perioperative complications associated with pancreas transplants. Additionally, these conditions are also connected with a decrease in the long-term viability and performance of the graft [10,11,12,13,14,15,16]. Because of this clinical evidence and to minimize the duration of CIT as much as possible, SPKTs are regularly conducted as urgent surgical procedures, including emergency operations at night. 

So far, trials analyzing the effect of the timepoint of starting transplant procedures in renal and liver transplant settings have had conflicting results, whereas, to our knowledge, data in the setting of pancreas transplantation in ET region are missing [17,18,19,20,21,22,23,24,25,26,27]. 

To bridge the significant evidence gap, we carried out a retrospective analysis at a single center, encompassing all patients who received SPKTs between 1998 and 2018. This study aimed to assess how the start time of the transplantation influenced surgical performance, graft function, patient survival, and outcomes, in addition to examining the frequency of perioperative complications.

## 2. Methods

### 2.1. Study Design and Study Population

We conducted a retrospective review of medical records for all adult patients who underwent SPKT at the University Hospital of Leipzig from 1998 to 2018. The information was sourced from an electronic clinical database that was assembled in a prospective manner. This study concentrated on the time of transplant procedures (daytime versus nighttime) regarding perioperative complications as well as allograft and patient function and outcome. To compare daytime and nighttime surgical procedures, the participants of this study were categorized into two cohorts according to the start time of SPKT. Surgical operations commencing from 8 a.m. to 6 p.m. were classified as daytime procedures, while those beginning from 6 p.m. to 8 a.m. were categorized as nighttime procedures. This study received ethical approval from the local ethics committee (AZ: Nr: 111-16-14032016). The study excluded individuals under 18 years of age, recipients of only kidney transplantation (KTA), patients undergoing pancreatic re-transplantation, and cases with incomplete or unavailable data regarding the procedure’s start time.

### 2.2. Outcome Analysis

We examined all pertinent parameters related to the recipient, donor, and outcomes in relation to the time of day the transplant surgery commenced. Standard characteristics of the study population prior to transplantation encompassed common parameters for recipients and donors, including age, gender, body mass index (BMI), reasons for donor death, pancreas donor risk index (pDRI), and type of offered transplant organs (locally procured versus imported), as well as the donor’s health conditions and hospital journey, such as the use of catecholamines, presence of arterial hypertension, and duration of stay in the intensive care unit (ICU-LOST). The collected data on recipients further included the length of time they had been diagnosed with diabetes mellitus, their time spent on the waiting list, the period in which they received dialysis before the transplant, and details on metabolic endocrine and lipid metabolism. They also covered specific comorbidities such as the existence of coronary heart disease and peripheral vascular disease (PVD), blood pressure levels, arterial hypertension, and the quantity of antihypertensive medications taken.

Data encompassing the perioperative and post-transplant phases were meticulously compiled to include key indicators of clinical progression. These encompassed the length of surgery, the volume of blood loss, and the durations of both cold and warm ischemia for the transplanted pancreas and kidney. Additionally, the frequency of perioperative complications, both surgical and nonsurgical, following SPKT, was documented for comprehensive analysis.

Within this framework, surgical complications are characterized as common issues associated with pancreas grafts, such as pancreatitis or pancreatic abscess, impaired graft function, simultaneous acute rejection, hemorrhage, leakage at the surgical connection, and graft thrombosis, all of which may necessitate subsequent surgical revisions or interventional treatments. 

Additionally, this study examined immunological factors and immunosuppressive conditions, including human leukocyte antigen (HLA) mismatches, cytomegalovirus (CMV) status, and induction therapy. It also assessed patient outcomes, long-term graft functionality, and survival rates. The research analyzed endocrine and lipid metabolism indicators such as the ratio of low-density lipoprotein (LDL) cholesterol to high-density lipoprotein (HDL) cholesterol, HbA1C percentage, and C-peptide levels in ng/mL. Renal function was evaluated through measurements of creatinine and urea in mmol/L, monitored for up to five years post-transplant.

Suspicions of acute rejection episodes arose when patients exhibited a rapid increase in serum amylase/lipase or glucose levels, a notable drop in serum C-peptide, or a rise in serum creatinine levels. These symptoms often presented alongside diminished urine production, abdominal discomfort, and ultrasound findings indicating graft enlargement. Confirmation of rejection was sought through endoscopic biopsies of the graft’s duodenal segment when possible, and kidney graft biopsies were performed as needed for verification. Pancreatic biopsy procedures were excluded from the methods used for diagnosis. For acute cellular rejection, treatment typically involved pulsed steroids or the administration of anti-thymocyte globulin (ATG) at a dose of 8 mg per kg body weight, alongside an escalation of baseline immunosuppression. In the case of humoral rejection, plasmapheresis and immunoglobulin therapy were employed [28]. DGF of the kidney is identified by the necessity for dialysis during the first week following the transplant [29]. Delayed graft function in the pancreas is characterized by the requirement for temporary insulin therapy from the initial postoperative phase up to the point of hospital discharge [30].

### 2.3. Surgical Methods/Anticoagulation/Immunosuppression Protocols

As previous sections noted, both pancreas and kidney transplants were performed adhering to the international protocols and standards set by Eurotransplant [15,31,32,33,34,35,36]. In summary, the pancreas was excised employing a no-touch method, remaining connected as a single piece with the spleen and duodenum. The superior mesenteric and splenic arteries underwent reconstruction with the donor’s iliac Y-graft prior to the intraperitoneal placement of the pancreas graft in the right iliac fossa. Usually, the arterial anastomosis was attached to the recipient’s common iliac artery, while the venous anastomosis (portal vein) was connected to the inferior vena cava. For exocrine secretion, a hand-sewn side-to-side duodenojejunostomy was performed 40 cm past the Treitz flexure [15,35]. Kidneys were implanted in the opposite iliac fossa with the vascular connections made to the external iliac vessels. The ureter was inserted into the bladder using the Lich–Gregoir method, supported by a double J catheter as an intraureteral stent [37]. The surgical core team for SPKTs at our center consisted of three experienced consultant surgeons during the investigated period, who were responsible for the SPTK procedures and were present according to their duty roster, with no differences between day- and nighttime presence between the three consultants. Organ harvesting and organ implantation were always performed by different teams. The local retrieval teams for organs coming from regionally proximate hospitals consisted of an experienced senior consultant in visceral surgery with a requirement of retrieval of at least 20 pancreata before going on duty for organ retrieval. The anesthesiologic teams did not differ between day- and nighttime surgery; at our center, there was always an anesthesiologic consultant present during every procedure.

According to our anticoagulation protocol, a bolus of 5000 IE of unfractionated heparin (UFH) was administered intraoperatively shortly before vessels clamping, followed by a postoperatively started UFH perfusor during the first 7 postoperative days with an intended PTT of 40–50 s, combined with ASS 100 mg/d. 

The immunosuppression approach entailed an initial phase of induction therapy, which was then followed by a triple maintenance therapy protocol as previously outlined. The induction phase utilized therapeutic agents such as anti-thymocyte globulin (Thymoglobulin) or the interleukin-2 receptor antagonist basiliximab (Simulect^®^). The ongoing maintenance therapy included a combination of calcineurin inhibitors like Cyclosporin (Sandimmun Neoral^®^) or Tacrolimus (Prograf^®^), possibly in conjunction with antimetabolites such as Sirolimus (Rapamune^®^) or Mycophenolate Mofetil (MMF; Cell Cept^®^, Myfortic^®^), and a gradually decreasing dosage of steroids (Prednisolone^®^) [38,39].

### 2.4. Statistical Analysis

In the baseline data, continuous variables are represented as mean ± standard deviation, while categorical variables are shown as frequencies and percentages. To compare the study groups, suitable tests for statistical significance were employed, such as Student’s *t*-test, the chi-square (χ2) test, analysis of variance (ANOVA), the Kruskal–Wallis test, and the Wilcoxon–Mann–Whitney test. The main focus of our research was to evaluate how the timing of SPKT initiation (daytime vs. nighttime) affects the rate of perioperative graft-related complications that necessitate further surgical or interventional procedures. The secondary endpoint included patient and allograft failure/survival as well as graft function and outcomes following SPKTs. Within this study, the criteria for pancreas graft failure included the resumption of insulin treatment, pancreas removal, or the necessity for a subsequent transplantation. Similarly, kidney graft failure was characterized by the initiation of dialysis, kidney removal, or the requirement for a re-transplantation.

A logistic regression analysis was conducted to calculate the likelihood of perioperative complications related to surgery, considering one or several predictive factors. Included variables in the model are start time of the operation (nighttime/daytime), recipient characteristics including age, sex, body mass index > 30 kg/m^2^, time on dialysis pretransplant, smoking habits, comorbidities (peripheral arterial disease and cardiovascular disease), choice of immunosuppressive agents, donor factors including age, sex, BMI, pDRI, ICU-LOS, cardiac arrest, donor cause of death, duration of surgery, and cold and warm ischemia time, as well as surgical related factors including delayed renal graft function and biopsy-proven rejection. 

The multivariable model was built by performing a stepwise variable selection procedure including those presenting *p* < 0.05 in univariable analysis. Significant findings were reported as the odds ratio (OR) accompanied by a 95% confidence interval (CI) and the *p*-value from the likelihood ratio test. In the case of variables not chosen in the multivariable analyses, neither the *p*-value from the score test nor the OR values were disclosed.

A sequential Cox proportional hazards regression approach was used to calculate hazard ratios (HRs) with 95% confidence intervals (CIs) for assessing the link between the timing of interventions and secondary results (allograft survival/failure). The univariate analysis took into account whether it was night or day, along with the recipient’s age, gender, and body mass index, donor age, gender, and BMI, as well as transplant-related parameters including era of SPKT (1998–2006 and 2007 to 2017), implantation order (pancreas first versus kidney first), warm and cold ischemia times, and choice of immunosuppressive agents. For the multivariate analysis, a backward regression technique was utilized, incorporating clinically significant variables and those achieving a *p*-value < 0.05 in univariate analysis. Survival probabilities were estimated using the Kaplan–Meier estimator, while the log-rank test was utilized to determine statistical disparities among the cohorts. Graft survival was gauged from the initial transplant to graft loss, considering both patients who passed away with a working graft and those whose grafts remained operational at the conclusion of this study. Patient survival was calculated from the time of transplant to the patient’s demise, with adjustments made for those still living when the study ended. For recipients who were either alive or untraceable at their final known interaction, their survival duration was limited to the last known point. All statistical evaluations were conducted utilizing SPSS software (version 21.0, SPSS Inc., Chicago, IL, USA). A *p*-value of less than 0.05 was deemed to indicate statistical significance.

Please note that this study has conducted a partial analysis of data from a database that was prospectively gathered, containing information about candidates for transplantation and individuals who received a pancreas transplant. Portions of this data have been published in previous works. However, those publications addressed different inclusion criteria, tackled distinct questions (such as SPKTs for type 1 vs. type 2 diabetes mellitus, sequence of graft implantation, types of dialysis modalities, etc.), and involved various patient groups and time periods. In our current analysis, we focus on the impact of the timing of surgical procedures on both the immediate and extended outcomes for patients and graft functionality. These particular findings and data have not been previously published [36,37,38,39,40,41,42,43,44].

## 3. Results

### 3.1. Baseline Characteristics

**Figure 1:**
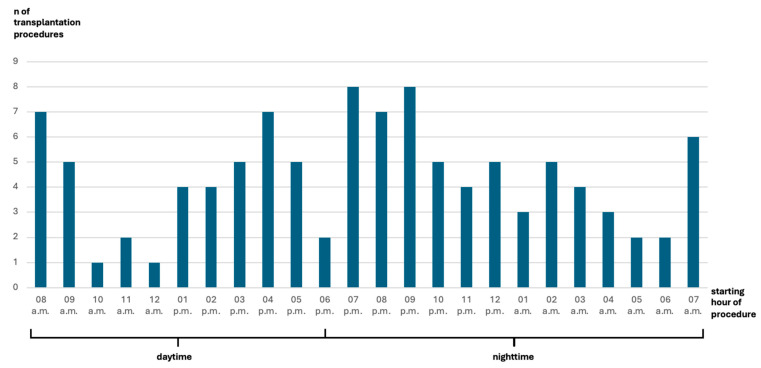
Bar graph illustrating number of procedures and distribution of start times.

Throughout the duration of this study, 105 simultaneous pancreas–kidney transplants were carried out. Among these, 43 transplants (41%) took place in the daytime, while the remaining 62 transplants (59%) occurred at nighttime (as shown in Figure 1).

The mean follow-up period of this study was 12.5 ± 2.8 years. Baseline demographic and clinical–pathological characteristics of donors and recipients according to the start time of transplantation used in our study are illustrated in Table 1. The average age and gender ratio of recipients were comparable between daytime and nighttime SPKTs. Further, no differences were observed regarding donor age, gender, BMI, comorbidities, offered organs (locally procured versus imported), or recipient comorbidities. There were no statistically significant differences between the sequence of the graft implantation order according to day- and nighttime procedures (*p* = 0.07) (Table 2).

Induction therapy was conducted in 38 (88%) patients in the daytime group and in 56 patients (90%) in the nighttime group; the number of HLA mismatches was also identical between both groups. 

The mean CIT of pancreas and kidney was also similar in both groups, with 11.2 ± 2.6 and 12.3 ± 3.2 h in daytime procedures compared to 10.9 ± 2.5 and 11.6 ± 2.7 h in nighttime procedures (*p* = 0.667 and 0.411). The duration of anastomosis was consistent regardless of the time of day the operation was performed, with an average time of 37.4 ± 8.5 min for pancreas and 34.8 ± 7.2 min for kidney transplant procedures in the daytime group compared to 38.9 ± 10.2 and 38.1 ± 11.9 min in the nighttime group (*p* = 0.664 and *p* = 0.221). The overall duration of the surgical procedure for simultaneous pancreas–kidney transplants (SPKTs), from the initial skin incision to the final wound closure, was notably less during the night than during the day (369 ± 92 min versus 405 ± 111 min; *p* = 0.04). 

### 3.2. Peri- and Postoperative Outcome/Complications

No statistically significant differences in the risk of typical pancreas transplant-related surgical and infectious complications were observed when nighttime operations were performed compared to daytime surgery (Table 3). Rates of pancreatitis, organ thrombosis, bleeding, and anastomic leak as well as viral and bacterial infections were comparable between both groups. Complications from surgery necessitating a reoperation or interventional treatment occurred in 21 (34%) nighttime procedures and 17 (40%) daytime procedures and were also not statistically relevant. 

Acute rejection episodes were observed in 13.3% of cases in total, with 11.6% in daytime and 14.5% in nighttime recipients. The timing of the surgery appears to correlate with the occurrence of DGF. Transplants performed at night resulted in DGF in fifteen instances (24.1%), in contrast to only four cases (9.3%) from surgeries conducted during the day. However, this pattern did not achieve statistical significance, with a *p*-value of 0.06, as shown in Table 3.

Factors that were statistically significant (with a *p*-value less than 0.05) as risk determinants for complications related to surgical pancreas grafts in the analysis using univariable logistic regression included recipient BMI > 30 kg/m^2^, time on dialysis pretransplant, donor age, donor BMI, cerebrovascular cause of donor death, cardiac arrest of the donor, pDRI, and length of ICU stay of the donor, as well as cold ischemia time of the pancreas (Table 4). Factors showing a trend towards statistical significance were recipient peripheral vascular disease, HLA mismatches > 2, renal delayed graft function, and incidences of rejection episodes. In contrast, the operative start time of SPKT (daytime versus nighttime) and other notable factors increasing the risk for complications including recipient age, recipient smoking history, recipient cardiovascular disease, anastomoses time, and choice of induction therapy were not linked to the occurrence of surgical complications that required reoperation in either univariable or multivariable analysis. Similarly, the gender of the recipient and donor, offered organs (locally procured versus imported), and the surgery duration were not statistically associated with surgical pancreas graft-related complications.

Variables that emerged as potential indicators and predictors of surgical complications in the univariate analysis (with a *p*-value less than 0.05) were chosen for the multivariable Cox model analysis (Table 4). The factors that continued to show a heightened independent risk encompassed recipient BMI > 30 kg/m^2^, donor age, donor BMI, and cold ischemia duration of over 15 h. Other variables recognized in the univariable analysis did not maintain their significance as independent risk factors in the multivariable analysis.

### 3.3. Patient Survival and Graft Survival for Nighttime Compared with Daytime SPKTs

**Figure 2:**
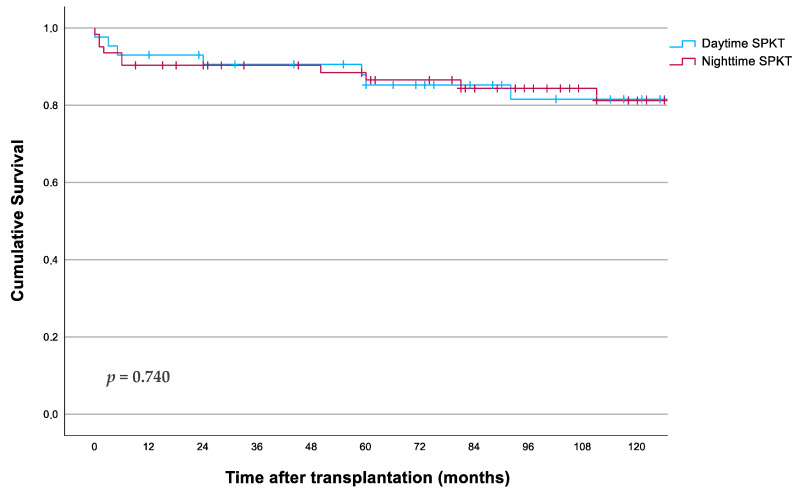
Patient survival according to starting timepoint of surgery.

Survival curves for both patient and pancreas and kidney graft survival were created using Kaplan–Meier analysis, comparing daytime and nighttime SPKTs. The survival of patients who received transplants at night was comparable to those who received them during the day, with one-year survival rates of 91% for nighttime and 93% for daytime recipients, and five-year survival rates of 86% and 85.2%, respectively (*p* = 0.816, Figure 2).

**Figure 3:**
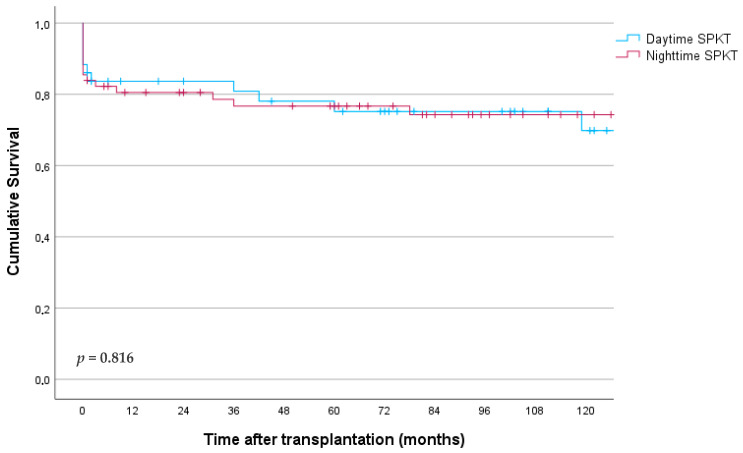
Pancreas graft survival according to starting timepoint of surgery.

The survival rates for both pancreas and renal grafts were similar, regardless of whether the transplants occurred during the day or at night. So, one-year pancreas graft survival was 83% and 81% for daytime and nighttime procedures, and 75% and 77% at 5 years, respectively (*p* = 0.912, Figure 3).

**Figure 4:**
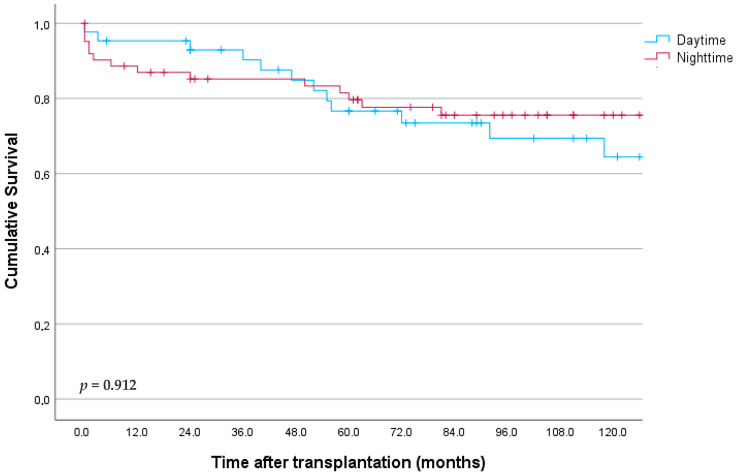
Kidney graft survival according to starting timepoint of surgery.

In contrast, one-year renal graft survival was 91% and 87% for daytime and nighttime procedures, and 76% and 80% at 5 years, respectively (*p* = 0.740, Figure 4).

The unadjusted Cox proportional hazard model demonstrated that the timepoint of SPKT start (daytime versus nighttime) had no statistically significant impact of three-month and 5-year pancreas graft survival (Table 5). The daytime patients had a 1.61 (95% CI: 0.68–3.7) hazard ratio for 3-month pancreas allograft failure and a 1.1 HR (95% CI: 0.5–2.39) for 5-year pancreas allograft failure. Additionally, our model and analysis revealed that the age of both the donor and recipient, the recipient’s BMI, and the length of CIT for the pancreas were independent factors predicting the likelihood of pancreas allograft failure within the first three months and up to five years post-SPKT. On the other hand, factors such as the recipient’s gender, the donor’s BMI and gender, whether the organs were procured locally or imported, the era of transplantation, and the sequence in which the grafts were implanted significantly influenced pancreas graft survival only at the three-month mark, without affecting the survival rate at the five-year milestone (Table 5). 

## 4. Discussion

As far as we are aware, this research offers the first analysis of results for SPKT recipients, categorized based on whether the surgery occurred during the day or night, across the Eurotransplant region. We showed that there are no significant differences in postoperative complications requiring reoperations or pancreas graft-related complications as well as no variance in the survival of patients and pancreas and renal grafts at the 1-year, 3-year, and 5-year benchmarks between the starting timepoint of operation. The positive outcomes we observed are promising for both patients and the transplant community, indicating that the safety measures and quality standards in place can guarantee the safety, consistency, and reliability of SPKT, regardless of the time the procedure begins.

Despite being an intricate procedure with a high degree of difficulty, simultaneous pancreas–kidney transplantation (SPKT) largely hinges on the quality of surgical performance. This is even more pronounced given the continued strides made in immunosuppressive treatment, as well as peri- and postoperative management, which have decreased the frequency of graft losses unrelated to surgery. While medical mishaps can arise from various factors, there is a strong body of evidence indicating that lack of sleep and physical exhaustion contribute significantly to the risk of surgical errors. Fatigue may lead to immediate short-term complications and consequences requiring prompt surgical correction, such as typical ischemia reperfusion injury (IRI)-related complications including graft infection, bleeding, or vascular issues. Moreover, it can result in delayed and later complications including rejection episodes and chronic infections [45]. 

With regard to graft function, we found a slightly non-significant higher rate of renal DGF in the group of nighttime surgery. The underlying causes for this finding have not been completely understood yet. One explanation could be that a higher rate of “non-optimal donors” were transplanted at night, though no significant differences in donor age or BMI between both groups were observed. 

Secondly, in some of the patients at our center, the kidney was transplanted before the pancreas, which happens slightly more often in the daytime group when two teams operate concurrently: one is responsible for the back-table preparation, while the other performs the transplantation operation. 

In a previous publication from our group, we showed enhanced pancreas graft survival when the kidney was implanted before the pancreas graft (kidney first, KF) in SPKT patients, hinting that the graft implantation sequence may show beneficial effects on the outcome in SPKTs in favor of the KF strategy. In our current publication, we found a slight, but non-significant, difference in graft implantation sequence between day- and nighttime procedures (*p* = 0.07). Therefore, we could not completely rule out that the implantation sequence might have influenced our results. To rule out this limitation completely, prospective studies comparing implantation sequence as well as day- vs. nighttime procedures and the effects of two teams working parallel in SPKT are necessary [36].

In our study population, we observed no increase in the number of (surgical) pancreas graft-related complications necessitating reoperations following simultaneous pancreas–kidney transplantations (SPKTs) performed at night compared to those conducted during the day. This finding stands in contrast with existing literature that links impaired surgical performance to sleep deprivation and physical fatigue. Methods utilizing simulation-based frameworks exist to quantitatively measure both psychomotor and cognitive abilities [46,47,48]. In their seminal work, Dawson et al. provided a compelling and easily understood measure of the impact of fatigue, finding that cognitive psychomotor capabilities diminish after 24 h of continuous wakefulness to a degree comparable to the impairment seen with a blood alcohol level of approximately 0.10% [49]. Similarly, research by Rothschild et al. suggests that sleeping less than 6 h in a 24 h period is associated with increased surgical complications [5]. These findings align with those of Grantcharov et al. and Taffinder et al., who employed models with laparoscopic simulators to show that surgeons exhibit a greater frequency of errors when performing procedures the morning after being on call [50,51].

Research in other surgical disciplines, such as orthopedics and colorectal surgery, has indicated a pattern where surgeries performed during the night are linked with a greater need for subsequent reoperations [26], or show a heightened risk of complications such as anastomotic leaks, with the starting time of the surgery being a significant independent risk factor [27]. 

In transplant medicine, a recent meta-analysis has uncovered an association between transplant procedures carried out outside of standard working hours and an increase in mortality rates for those transplants performed during off-hours [52]. Interestingly, however, multiple studies have reported that nighttime surgeries do not necessarily lead to worse outcomes, challenging the notion that fatigue inherently increases surgical risk [53,54].

Thus, we can only theorize why our study shows no disparity in complication frequencies between daytime and nighttime transplant procedures. A plausible explanation is our employment of dedicated transplant teams that boast both high surgeon caseloads and extensive collective experience, supported by the standardization of surgical protocols within our facility. Furthermore, issues of sleep deprivation and fatigue are not restricted to the surgical team but extend to the anesthetic team, operating room nurses, and the critical care staff, all of whom play crucial roles in the immediate postoperative period. Differentiating SPKT operations from other nighttime emergency interventions, the anesthetic procedures during the operation, as well as subsequent patient care, are typically overseen by a board-certified, experienced anesthesiologist, rather than by junior residents. It is possible that the high level of expertise of the attending physicians, which includes both the surgeons and the anesthesiologists, may mitigate the potential adverse impacts of fatigue [21,26].

Standard surgical protocols involve a series of specific maneuvers and steps which, when internalized and automated, necessitate less active cognitive engagement—particularly in a high-volume transplant center such as ours, with a limited number of surgeons performing these operations. Under nocturnal, sleep-deprived conditions, these routinized actions and procedural expertise are conducive to the optimal management of cognitive resources [55]. Moreover, it is likely that surgeons develop personal strategies to manage and mitigate fatigue over the course of their training [56,57]. Just as they refine their technical abilities, surgeons are trained to maximize their performance despite extended periods without sleep [24]. Consequently, it can be posited that the combination of training and experience serves to counterbalance the effects of fatigue [57].

Research in renal transplantation has indicated that the identity of the lead surgeon might introduce bias in studies examining the impact of operations conducted at night [21,26]. During the day, less experienced residents and fellows may participate in surgeries for training purposes, while surgeries at night might occur in the absence of a transplant consultant. Nonetheless, at our institution, all simultaneous pancreas–kidney transplantations (SPKTs) are performed by a selected and small group of highly skilled consultant transplant surgeons, a practice that remains constant regardless of when the surgery starts. Consequently, we can dismiss the notion of decreased experience during night hours as a confounding factor in our analysis.

In the setting of visceral organ transplantation, the literature presents a scant array of findings regarding the effects of day versus nighttime surgery. Within orthotopic liver transplantation (OLT), investigations of data from both the Eurotransplant region and the United Network for Organ Sharing (UNOS) database—specifically studies by Becker et al. and Orman et al.—have indicated no discernible variations in the survival rates of patients and grafts at intervals of 30 days, 90 days, and one year, or in perioperative complications when segmenting patients by the timing of their procedures [19,27]. Another study from the US conducted by Lonze et al., which encompassed 587 liver transplant recipients, noted that those who underwent nighttime transplants experienced marginally longer surgeries and required more blood products [58]. Despite these differences, the study observed no significant variations in perioperative complications between day and night procedures. However, it did uncover a doubling in the risk of early postoperative mortality, within seven days following the transplant, for those in the nighttime surgery cohort. Yet, it is worth noting that the long-term survival rates of patients were not influenced by the timing of their surgery [58].

Regarding nighttime transplant operations in other solid organ transplantation contexts, noteworthy contributions to the debate come from several studies. One of the most significant contributions on kidney transplants performed at night emanates from research led by Schrem et al. In their inventive approach, the team developed a risk-balancing score and recommended avoiding kidney transplants during the time frame from 3 a.m. to 6 a.m., provided that CIT could be kept under 23.5 h [20]. Echoing these sentiments, Fechner et al. scrutinized a dataset comprising 260 kidney transplant recipients and identified an increased risk of reoperation, particularly due to vascular complications following nighttime procedures, alongside an elevated risk of graft failure [25]. Montaigne et al. presented evidence suggesting that renal transplants benefit from daytime declamping, noting improved post-transplant survival rates that held irrespective of ischemia time and other confounders [59].

Conversely, Shaw et al. observed a decrease in vascular complications in kidney transplant patients who underwent surgery at night, without any noticeable differences in the survival of patients or grafts in comparison to those who had surgery during the day [23]. This perspective is corroborated by several other investigations [17,18,21,24,26,60,61] in the realm of renal transplantation surgery, where their findings reported no significant differences in complication frequencies or patient and graft survival rates between day- and nighttime surgeries. Remarkably, Van Brunschot et al., analyzing data from 4519 renal transplantations in the Dutch Organ Transplant Registry, posited potential advantages of nighttime surgeries on the rates of technical graft failure [21]. These varied outcomes highlight the complexity and diversity of findings related to operative timing in transplant surgery, underscoring the need for more nuanced, perhaps protocol-driven research to better understand the implications of nighttime transplantation procedures.

Typically, transplant centers globally base their daily decisions on a steady evaluation of risks between minimizing CIT and the possible impact of nighttime surgeries. It is an unquestionable principle in transplant medicine that reducing CIT is paramount, providing a compelling justification for conducting organ transplants at any hour. Based on previous findings and our own results, the risk of graft failure in pancreas transplantation rises to a relative risk of 1.0 for a pancreas preserved for <12 h, to 3.67 for 12–16 h CIT, and even further to 6.6 for 20–24 h of cold ischemia [11]. In accordance with some previous data, in our analysis, we found an association between CIT and increased surgical (technical) pancreas graft-related complications [10,11]. In contrast, other reports have produced conflicting data, finding no association between CIT and the risk of surgical complications [62,63].

Therefore, in our eyes, postponing the recipient procedures appears disadvantageous in the case of SPKTs for optimal pancreas transplant function and outcome. The timing of the transplant largely depends on the availability of the donor organ and the timing of its procurement. It might be proposed to delay the procurement of the organ. Yet, this may lead to additional deterioration of the organs due to the extended impacts of brain death and would also heighten financial and logistical strains on donor hospitals, a common issue across many Eurotransplant (ET) region countries [64]. Moreover, the current systems for allocating organs lead to extended transport durations. This requires, first, the swift transplantation of the organ once it arrives, and second, a reduction in potential delay-causing factors, such as prolonged storage at the airport, scarcity of operating rooms and anesthesia services, and complex surgical dissections.

Our findings offer initial proof within the ET region that nighttime surgeries do not adversely affect the immediate or extended success and longevity of grafts post-SPKT. In addition to similar short-term results observed in our group, such as reoperations due to complications with the pancreas graft, operations conducted at night did not correlate with an increase in the rates of re-transplantation, delayed graft functioning, or confirmed rejections through biopsy. Further, short and long term, both patient and graft survival were also comparable between both groups. This additionally confirms the established safety protocols that are implemented at our transplant center. As a result of our findings, predicting the risk of surgical complications by identifying a limited set of risk factors (recipient BMI, donor age and donor BMI, preservation time) may assist in optimal donor and recipient selection and appropriate and careful risk prediction and stratification in donor/recipient selection and is helpful in risk factor modulation for future pancreas transplantation.

The data showcased in this study clearly indicate that SPKTs must be conducted regardless of the time of day to reduce CIT effectively and keep it as short as possible, particularly in IRI-sensitive organs such as liver and pancreas—where the ischemia time is very important for transplant-related outcome and function—and thus maximize transplant outcomes. While the data did not show a correlation between nighttime operations and poorer results, it stands to reason that well-rested staff would be advantageous. As a result, additional strategies should be implemented to counteract any possible negative impacts of nighttime surgeries in the field of transplant medicine. Among the most promising approaches is machine perfusion, which could potentially extend organ preservation times beyond the capabilities of traditional static cold storage and help reduce and improve the impacts of IRI [65]. In the realm of kidney and liver transplants, employing this technique to perfuse “marginal organs” aims to enhance organ utilization rates from marginal donors with acceptable outcomes, thus expanding the pool of viable donor organs [66,67,68]. While prolonging CIT and potentially delaying the start time of surgeries might be feasible for kidney and liver transplants through machine perfusion, this strategy currently seems impractical for pancreas transplantation. The pancreas is particularly susceptible to edema and ischemic damage during retrieval and preservation, which can lead to microcirculatory dysfunction, likely a major reason why pancreatic perfusion has not attracted as much attention as it has for other organs previously [69,70,71]. Nevertheless, recent experimental and initial human trials of pancreatic machine perfusion look promising [69,72,73,74].

Several important limitations warrant discussion regarding the present study.

First, the relatively small sample size distributed across each group and the study’s retrospective, non-randomized nature must be acknowledged. Given these constraints—specifically the limited patient numbers per analysis subgroup—the findings should be regarded prudently; a direct clinical application is inadvisable due to the potential for low statistical robustness and the absence of established causal relationships. Nevertheless, the primary contribution of retrospective analyses lies in the formation of hypotheses that could be substantiated through future prospective research.

Second, the categorization of surgeries as either daytime or nighttime is inherently arbitrary and can introduce classification bias. Our time stratification, where daytime is defined as the period from 8 a.m. to 6 p.m., while nighttime is designated as the interval from 6 p.m. to 8 a.m., aligns closely with our hospital’s standard working hours (7:30 a.m. to 5:30 p.m.), and we hypothesize that surgeries scheduled early in the morning (for example, at 6:30 a.m.) are likely completed by the overnight staff. This particular division into ten- and fourteen-hour segments was adopted to ensure a sufficient number of subjects for robust multivariate statistical analysis. Even so, when exploring alternative time divisions (day: 6 a.m. to 6 p.m.; night: 6 p.m. to 6 a.m.), the results remained consistent. Nonetheless, an omission in our data includes the timing of organ procurement. Existing literature on renal and liver transplants indicates that daytime procedures may frequently utilize organs procured at night, which could introduce another element of bias to our retrospective examination [19].

Further complicating factors include the timing of operations in relation to weekends or holidays, which may also bias results. In renal transplantation, one transplant group has recently noted that weekend surgeries pose an increased risk for surgical complications [36]; however, it was observed that this factor did not adversely affect the one-year outcome in liver transplant recipients [37]. Concerning further possible confounders, to our knowledge the retrieval teams for organs were identical for day- and nighttime SPKT.

Lastly, evaluation of BMI certainly does not depict an optimal parameter to assess nutritional status in patients with chronic renal insufficiency, diabetes mellitus, and renal replacement therapy. However, based on the retrospective design of our analysis, no alternative nutritional score is available during our study period of 20 years, nor was it possible to evaluate for instance sarcopenia scores using CT images to assess the nutritional status of the investigated patients.

## 5. Conclusions

To summarize, our data gleaned from a retrospective analysis at a single German transplant center within the Eurotransplant domain suggest that simultaneous pancreas–kidney transplantation (SPKT) is a procedure whose safety is not compromised by the timing of its initiation. Our findings indicate that nighttime SPKT does not carry an augmented risk of perioperative surgical or infectious complications when juxtaposed with its daytime counterpart. Additionally, neither patient nor graft survival appears to be influenced by the operative time. The rate of surgical (technical) complications following SPKT increased with prolonged CIT, recipient BMI > 30 kg/m^2^, and elevated donor age and BMI. Taking this together, appropriate and careful risk prediction and stratification in donor/recipient selection and consecutive risk factor modulation is especially relevant for the short- and long-term success of pancreas transplantation. Thus, the imperative to minimize cold ischemia time (CIT) and thereby enhance transplant outcomes necessitates that SPKT procedures be undertaken as emergencies, transcending temporal boundaries. In light of promising findings, alongside progress in machine perfusion (MP) techniques in kidney and liver transplants, further research is pivotal in pancreatic MP. Such investigations could refine pancreas graft quality assessments and potentially expand the donor pool, a measure that is particularly necessary for the clinical milieu.

## Figures and Tables

**Table 1:** Baseline perioperative transplant characteristics of recipient and donor with SPKTs according to operative starting time (daytime or nighttime).

Variables	Daytime (*n* = 43)	Nighttime (*n* = 62)	*p*-Value
Donor			
Age, years	25.1 ± 12.8	26.8 ± 11.4	0.389
BMI, kg/m^2^	22.5 ± 3.5	22.3 ± 3.4	0.597
Catecholamine use	28 (65)	38 (61)	0.691
Stay on intensive care unit, days	3.7 ± 3.1	3.2 ± 2.9	0.398
Cardiac reanimation	4 (9.3)	5 (8.1)	0.821
Hypertension, n (%)	5 (11)	6 (9.6)	0.784
Pancreas donor risk index	1.43 ± 0.03	1.56 ± 0.04	0.090
Locally procured versus imported organs	18 (42)/25 (58)	23 (37)/39 (63)	0.622
Recipient			
Age, years	42.8 ± 9.2	43.7 ± 8.9	0.711
Gender, male/female	22 (51)/21 (49)	36 (58)/26 (42)	0.484
BMI, kg/m^2^	24.4 ± 4.2	25.4 ± 3.8	0.856
HbA1c, (%)	7.8 ± 1.6	7.9 ± 1.9	0.386
Duration of diabetes, years	26.6 ± 8.4	27.7 ± 8.9	0.602
Comorbidities			
Cardiovascular disease, n (%)	14 (33)	17 (27)	0.573
Peripheral vascular disease, n (%)	6 (14)	11 (18)	0.604
Hypertension, n (%)	34 (79)	50 (81)	0.843
Number of antihypertensive medications	2.8 ± 1.3	2.5 ± 1.8	0.321
Previous dialysis, n (%)	33 (77)	47 (76)	0.912
Duration of dialysis, months	31.9 ± 7.8	34.7 ± 9.3	0.421
Waiting time, months	6.9 ± 6.3	9.8 ± 13.4	0.204
Renal replacement therapy			
Hemodialysis versus peritoneal dialysis	34 (79)/9 (21)	12 (19)/50 (81)	0.843
Transplant characteristics			
CMV D+/R−	7 (16)	13 (21)	0.621
HLA mismatches > 2/6	28 (65)	47 (75)	0.234
Immunosuppression			
Induction therapy (ATG/IL-2 RA/None)	30 (70)/8 (19)/5 (11)	39 (63)/17 (27)/6 (10)	0.576
Cold ischemia times, hours			
Pancreas	11.2 ± 2.6	10.9 ± 2.5	0.667
Kidney	12.3 ± 3.2	11.6 ± 2.7	0.411
Warm ischemia times, minutes			
Pancreas	37.4 ± 8.5	38.9 ± 10.2	0.664
Kidney	34.8 ± 7.2	38.1 ± 11.9	0.221
Operating time, minutes	405 ± 111	369 ± 92	0.040

Table legends: BMI, body mass index; HbA1c, glycosylated hemoglobin; CMV, cytomegalovirus; HLA, human leukocyte antigen; ATG, anti-thymocyte globulin; IL-2 RA, interleukin-2 receptor antagonist; SPKT, simultaneous pancreas–kidney transplantation.

**Table 2:** Graft implantation order according to day- and nighttime procedures (Chi-square; *p* = 0.07).

Graft Implantation Order	Day (8 a.m.–6 p.m.)	Night (6 p.m.–8 a.m.)
kidney first (KF)	23 (53%)	21 (34%)
pancreas first (PF)	20 (47%)	41 (66%)

**Table 3:** Intra- and postoperative outcome, function, and complications following simultaneous pancreas–kidney transplantation (SPKT) according to start time of transplantation (daytime or nighttime).

Variables	Daytime (n = 43)	Nighttime (n = 62)	*p*-Value
Pancreatitis/abscess (%)	6 (14)	8 (13)	0.865
Reoperation rates (%)	17 (39.5)	21 (33.9)	0.552
CMV infections	8 (18.6)	13 (21)	0.765
Delayed renal graft function (%)	4 (9.3)	15 (24.1)	0.060
Bleeding	4 (9.3)	7 (11.3)	0.693
Vascular thrombosis pancreas	4 (9.3)	6 (9.7)	0.943
Combined acute rejection episodes	5 (11.6)	9 (14.5)	0.621
Anastomic leak	1 (2.3)	1 (1.6)	0.792
Wound infections	18 (41)	20 (32)	0.313

Table legends: CMV, cytomegalovirus; SPKT, simultaneous pancreas–kidney transplantation.

**Table 4:** Logistic regression model for predictors associated with the risk of occurrence of (surgical) pancreas graft-related complications.

	Univariate		Multivariate	
Variables	Odds Ratio (95% CI)	*p*-Value	Odds Ratio (95% CI)	*p*-Value
Operative start time (nighttime versus daytime)	0.73 (0.32–1.64)	0.443		
Recipient characteristics				
Recipient age, years	1.03 (0.98–1.07)	0.267		
Recipient gender (female versus male)	1.14 (0.71–1.72)	0.301		
Recipient BMI > 30 kg/m^2^	20.90 (2.52–172.3)	<0.01	16.6 (1.9–148.12)	0.012
Time on dialysis pretransplant (per 1-year increase)	0.98 (0.96–0.99)	0.04		
Smoker (yes versus no)	0.75 (0.51–1.09)	0.213		
Recipient peripheral vascular disease (yes versus no)	1.34 (0.46–3.91)	0.08		
Recipient cardiovascular disease (yes versus no)	0.83 (0.34–2.1)	0.679		
Renal replacement therapy (peritoneal versus hemodialysis)	1.35 (0.43–4.27)	0.587		
Donor characteristics				
Donor age (years)	1.05 (1.01–10.09)	0.01	1.04 (1.01–1.09)	0.044
Donor BMI, kg/m^2^	1.21 (1.06–1.39)	<0.01	1.31 (1.1–1.54)	<0.01
Donor gender (female versus male)	0.57 (0.25–1.32)	0.192		
Donor cause of death (non-trauma versus trauma)	1.52 (1.04–2.23)	0.031		
Donor cardiac arrest (yes versus no)	7.7 (1.51–39.29)	0.014		
Duration of surgery, hours	1.01 (0.99–1.05)	0.600		
Imported versus locally procured organs	0.99 (0.39–2.52)	0.534		
Pancreas donor risk index (pDRI)	1.79 (1.32–2.6)	<0.01		
Donor stay length in ICU, days	1.19 (1.01–1.33(	0.032		
Cold ischemia time of pancreas, hours	1.23 (1.06–1.428)	<0.01		
Cold ischemia time of pancreas, >15 h	7.7 (2.2–26.1)	<0.01	8.05 (2.15–30.29)	<0.01
Anastomosis time pancreas (minutes)	1.007 (0.959–1.056)	0.791		
Immunosuppression				
Induction therapy				
No		0.737		
ATG	0.60 (0.17–2.17)	0.437		
IL-2-RA	0.68 (0.16–2.81)	0.593		
HLA mismatch >2 versus <2	1.27 (0.83–1.93)	0.09		
Transplant-Related Complications				
Delayed renal graft function (yes versus no)	0.67 (0.17–2.06)	0.09		
Rejection episodes (yes versus no)	2.8 (0.99–7.49)	0.053		

**Table 5:** Logistic regression analysis of predictors for pancreas allograft failure following simultaneous pancreas–kidney transplantation (SPKT).

Variables	Time after SPKT											
	3 Months						5 Years					
	Univariate Analysis			Multivariate Analysis			Univariate Analysis			Multivariate Analysis		
	HR	95% CI	*p*-Value	HR	95 CI	*p*-Value	HR	95 CI	*p*-Value	HR	95 CI	*p*-Value
**Donor**												
Age *	1.09	1.02–1.13	0.002	1.05	1.01–1.98	0.012	1.06	1.02–1.09	0.003	1.061	1.03–1.11	0.001
Gender (male vs. female)	1.37	0.58–3.25	0.251				3.7	1.02–8.45	0.145			
BMI *	1.16	1.02–1.35	0.032	1.24	1.07–1.42	0.003	1.16	1.02–1.35	0.026	1.11	0.92–1.31	0.174
Imported vs. locally offered organs	0.88	0.76–1.02	0.192				0.98	0.93–1.02	0.251			
**Recipient**												
Age *	1.06	1.01–1.13	0.013	1.10	1.03–1.18	0.004	1.08	1.02–1.14	0.008	1.06	1.011–1.12	0.018
Gender (male vs. female) *	0.33	0.15–0.97	0.036	0.24	0.08–0.70	0.008	0.58	0.25–1.31	0.07			
BMI *	1.17	1.06–1.31	0.001	1.23	1.09–1.39	0.008	1.20	1.01–1.35	<0.001	1.26	1.06–1.41	0.005
**Transplant**												
Era (1998–2006 vs. 2007–2018) *	4.8	1.1–21.14	0.035	7.1	1.5–33.5	0.013	2.11	0.86–625	0.089			
Implantation order graft	3.15	1.05–9.50	0.040	4.17	1.35–12.85	0.013	2.09	0.82–5.29	0.110			
(pancreas first vs. kidney first) *												
Start time of surgery (nighttime versus daytime)	0.622	0.26–1.46	0.277				0.91	0.41–1.98	0.432			
Warm ischemia time												
Pancreas	0.996	0.94–1.07	0.821				0.88	0.25–1.97	0.453			
Kidney	1.03	0.98–1.09	0.231				0.99	0.95–1.08	0.856			
CIT, hours												
Pancreas *												
0–8	Ref		0.002	Ref		0.004	Ref		0.02	Ref		0.06
8–15	0.61	0.1–12.4	0.129	0.58	0.05–0.86	0.01	5.18	0.61–43.4	0.131	2.98	0.6–14.9	0.183
>15	3.7	1.1–13.1	0.04	8.5	1.3–114.9	0.02	11.3	1.5–86.3	0.019	5.38	1.21–23.7	0.027
Kidney *												
0–8	Ref		0.07				Ref		0.012	Ref		0.008
8–15	0.46	0.2–8.9	0.58				0.13	0.02–0.99	0.048	0.38	0.1–1.6	0.07
>15	3.89	0.21–34.8	0.18				1.82	0.33–8.02	0.164	1.1	0.82–8.8	0.451
**Immunosuppression**												
Induction therapy												
None	Ref.		0.791				Ref.		0.342			
ATG	0.63	0.16–2.58	0.527				0.78	0.21–2.96	0.722			
IL-2 RA	0.72	0.19–2.91	0.791				1.03	0.27–4.12	0.961			

Table legends: BMI—body mass index; CIT—cold ischemia time; ATG—anti-thymoctye globulin; IL-2 RA, interleukin-2 receptor antagonist. * included in multivariate analysis

## Data Availability

Our database contains highly sensitive data that may reveal clinical and personnel information about our patients and lead to their identification. Therefore, according to organizational restrictions and regulations, these data cannot be made publicly available. However, the datasets used and/or analyzed in the current study are available from the corresponding author upon reasonable request.

## Abbreviations

ATG	anti-thymocyte globulin
BMI	body mass index
CI	confidence interval
CIT	cold ischemia time
CMV	cytomegalovirus
DGF	delayed graft function
ET	Eurotransplant
HDL	high-density lipoprotein
HLA	human leukocyte antigen
HR	hazard ratio
KTA	kidney transplantation alone
ICU-LOS	intensive care unit length of stay
IRI	ischemia reperfusion injury
LDL	low-density lipoprotein
MP	machine perfusion
OLT	orthotopic liver transplantation
OR	odds ratio
pDRI	pancreas donor risk index
PVD	peripheral vascular disease
SPKT	simultaneous pancreas–kidney transplantation
UNOS	United Network for Organ Sharing
US	United States
